# Research progress on the protective effects of aucubin in neurological diseases

**DOI:** 10.1080/13880209.2022.2074057

**Published:** 2022-05-28

**Authors:** Ping Yang, Qiaoyue Zhang, Hengyan Shen, Xinyu Bai, Ping Liu, Tao Zhang

**Affiliations:** a Department of Laboratory Medicine, Affiliated Hospital of Zunyi Medical University, Zunyi, China; b Department of Clinical Pharmacy, Key Laboratory of Basic Pharmacology of Guizhou Province and School of Pharmacy, Zunyi Medical University, Zunyi, China

**Keywords:** Pharmacological effects, molecular mechanism, Eucommia ulmoides Oliv

## Abstract

**Context:**

Aucubin (AU), an iridoid glycoside that is one of the active constituents of *Eucommia ulmoides* Oliv. (EUO) (Eucommiaceae), a traditional Chinese medicine, has been extensively studied in the management of neurological diseases (NDs). However, a comprehensive review of its effects and mechanisms in this regard is currently not available.

**Objective:**

To compile the protective effects and mechanisms of AU in NDs and provide a basis for further research.

**Methods:**

We used ‘aucubin’ as the ‘All Fields’ or ‘MeSH’ in PubMed, Web of Science and China National Knowledge Infrastructure without any limitation to search all relevant articles as comprehensively as possible; we selected the articles on AU treatment of NDs for summary.

**Results:**

Studies reviewed herein reported that AU improved the symptoms or prognosis of Parkinson’s disease, Alzheimer's disease, intracerebral haemorrhage, diabetic encephalopathy, epilepsy, anxiety and depression, and traumatic brain injury. The pharmacological mechanisms involved in repairing neuronal loss were postulated to include increasing γ-aminobutyric acid (GABA) content in the synapse, promoting differentiation of neural precursor cells into GABAergic neurons, providing antioxidant and anti-neuroinflammation activities, as well as enhancing autophagy and anti-apoptotic actions.

**Discussion and conclusions:**

The protective effects of AU on some NDs have been confirmed. According to the pharmacological effects, AU is also highly likely to have protective effects on other NDs, which can be realized by further *in vivo* and *in vitro* basic research, and clinical trials. In the future, AU may be used for clinical prevention or treatment of patients with neurological diseases.

## Introduction

Neurological diseases (NDs) are a serious public health problem, with sensory, motor, consciousness, or autonomic nervous dysfunction manifesting as chronically progressive and irreversible conditions. NDs, such as cerebrovascular, neurodegenerative, and mental disorders rank among the leading causes of disability-adjusted life years (DALYs) and death worldwide. Furthermore, the medical economic burden from the aforementioned disorders has rapidly increased since 1990. In China, the DALYs ratio of NDs increased by 7.4% from 1990 to 2015 (GBDNDC 2017). Treatment of NDs is critical to increasing and improving the lifespan and quality of life of patients, respectively, and mainly relies on the long-term use of medication. However, effective therapeutic drugs and methods are not readily available (Wang H 2019); furthermore, there are basically no treatment options that provide the patients with permanent or continuous relief. Consequently, there is a need to identify effective medicines to treat NDs (Zhang et al. 2018). Traditional Chinese Medicines (TCMs) have been used for more than 2,000 years and have gained widespread clinical application over this period. TCMs are composed of many different compounds with various structures and functions, with all the components acting in synergy on multiple targets, which may be the reason for their ability to treat complex diseases. In general, the clear and irreplaceable advantages of TCMs can be aligned with the disease and treatment complexity of NDs, which would be a pragmatic direction for new drug discovery.

In TCM, NDs refer to pathogenic factors, such as the six external factors (wind, cold, heat, dampness, dryness, fire), seven emotional factors (joy, anger, melancholy, brooding, sorrow, fear and shock), and trauma that invade the brain, causing disease related to apraxia of consciousness or disturbance in the functional activities of the central nervous system, and then lead to the disturbance in mental, action, and consciousness owing to decline in brain function. Its pathogenesis is closely related to the viscera, especially the kidneys. The kidneys yield and deposit an ‘essence,’ which modulates the brain. Cognitive function decline occurs due to lack of ‘energy’ in the brain, which is caused by the lack of the ‘essence’ in the kidneys (Li et al. 2006; Shi et al. 2018). The Yellow Emperor's Inner Canon, a Chinese medical classic, put forward the view that ‘memory, wisdom, and skill all come from kidney essence.’ The brain, nourished by the plentiful ‘essence,’ contributes to normal neuronal function, provides for strong memory, smartness, responsiveness, and invigorates the body, while the subdued function of the brain is induced by a decrease in ‘essence.’ A report from the clinical experience of professor HE Xingwei suggests that ‘tonifying the kidney’ is an effective and safe measure for stroke patients with motor impairment of the trunk, which is also classified as a ND (Peng et al. 2017). In conclusion, tonifying the kidney is an effective way to treat NDs.

**Figure 1.**
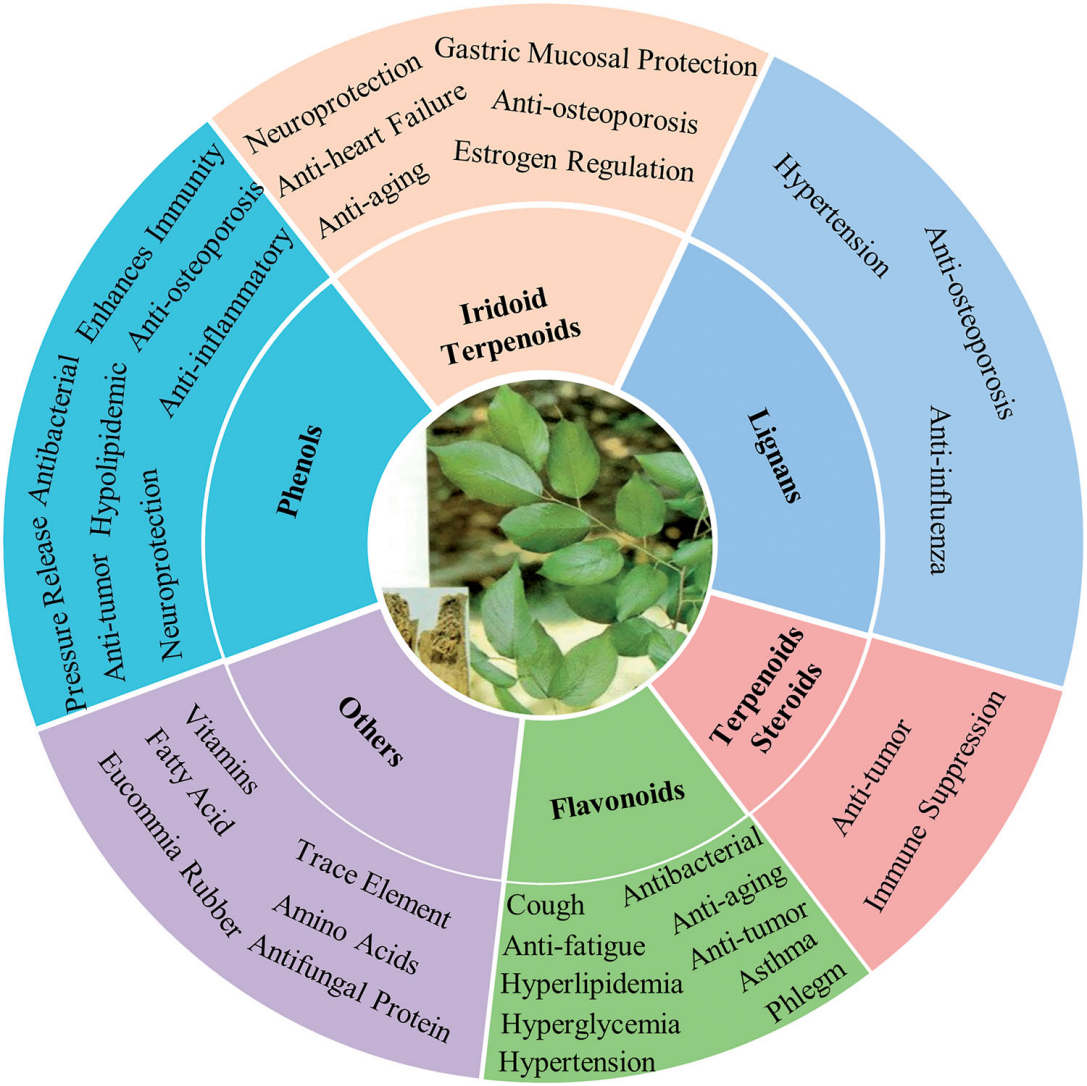
The main ingredients and corresponding pharmacological effects of EUO. The higher the content of ingredients, the greater the proportion.

*Eucommia ulmoides* Oliv. (EUO) (Eucommiaceae), a traditional Chinese herb, is one of the representative medicines for ‘tonifying the kidney,’ with a long history of use of more than 2,000 years. Bioactive chemicals of EUO include lignans, iridoids, phenols, and steroids, which play effective roles in processes such as nourishing the kidneys, neuroprotection, and regulating blood pressure. The active ingredients and corresponding pharmacological effects of EUO are shown in Figure 1.

In modern pharmacology, the activities of EUO against NDs have garnered much attention (Kwon et al. 2011; Fan et al. 2020). The composition of extracted bioactive molecules varies based on the different functional parts (leaves, seeds, bark, and staminate flower) and planting models (Li et al. 2015); nevertheless, the iridoid constituents are always abundant in any extract (Li et al. 2015). Aucubin (AU) (CAS: 479-98-1), also known as eucommia glucoside, is a representative component of iridoids of EUO with neuroprotective properties, has a molecular formula of C_15_H_22_O_9_, a relative molecular mass of 346.331, and the chemical name β-D-glucopyranose. AU can be extracted from the bark, leaves, fruits, or male flower of EUO; with the content being reported to reach 11.51%, which was more than chlorogenic acid and flavonoids (Zhu and Sun 2018). In addition, it has been noted that the role of AU in protecting against NDs is a promising resource for future treatment (Zhu et al. 2018; Zeng et al. 2020). With intensive research into its role and mechanism(s) of action, the effects of AU against NDs have been gradually discovered and confirmed. AU is the representative active component of EUO of the kidney-tonifying Chinese medicine, and it also has neuroprotective effects. This study summarizes its protective effects on NDs in order to provide references for subsequent research.

We used ‘aucubin’ as the ‘All Fields’ or ‘MeSH’ in PubMed, Web of Science and China National Knowledge Infrastructure, without any limitation on searching all relevant articles as comprehensively as possible. The articles on AU treatment of NDs were selected for summary, and shown main contents in Table 1. This study hopes to provide a basis for further research on the protective effects of AU on NDs by summarizing the existing research on AU in the treatment of NDs and its mechanisms. This not only allows readers to directly understand the current status of the neuroprotective effects of AU, but also provides direction for researchers to determine their next research.

**Table 1.** Summary of key information from the existing literature on AU for the treatment of NDs.

Authors, Year, Country	Type of NDs	Model/Animal/Cells	Doses of AU	Pharmacological activity	Biological analysis
Chen et al. 2019 China	Epilepsy	Li-pilocarpine-induced epileptic male ICR mice	50, 100 mg/kg	Anti-neuroinflammatory; Regulate neurotransmitters	Inhibit activation of hippocampal astrocytes and microglia; GABA
Chu et al. 2020 China	Anxiety and depression	Male Kunming mice	10, 20, 40 mg/kg	Regulate neurotransmitters	glutamate, noradrenaline, serotonin and GABA
Kim et al. 2014 Korea	–	*In vitro*: hippocampal neural stem cells *In vivo*: injury model of the rat sciatic nerve	*In vitro*: 1, 10 µg/mL *In vivo*: 2.5 mg/kg	Improve nerve regeneration	–
Li et al. 2021 China	–	Hydrogen peroxide (H2O2) – induced injury in SH-SY5Y cells	10^–6^, 10^–5^, 10^–4^ M	Anti-oxidative	Nrf2/HO-1pathway
Liu et al. 2015a China	Cerebral haemorrhage	Autologous blood was injected into the rat caudate nucleus to prepare cerebral haemorrhage model	4.2 mg/kg	Anti-neuroinflammatory	IL-1β and NF- κB expression
Liu et al. 2015b China	Cerebral haemorrhage	Autologous blood was injected into the rat caudate nucleus to prepare cerebral haemorrhage model	4.2 mg/kg	Anti-neuroinflammatory	TNF- α expression
Song et al. 2018a Korea	–	Differentiated neuronal cells and proliferating neural precursor cells (NPCs)	0, 10, 50, 100, 200, 400 µg/mL	Promote cell survival in differentiated, GABAergic and glutamatergic neurons; but reduce it in proliferating NPCs	–
Song et al. 2018b Korea	–	NPCs cultured from the rat embryonic hippocampus	0.01, 10 µM	Promote differentiation of neural precursor cells into GABAergic neurons	–
Wang et al. 2020 China	Traumatic brain injury (TBI)	*In vitro*: H_2_O_2_-induced primary cortical neurons *In vivo*: TBI model induced by weight drop	*In vitro*: 50, 100, 200 μg/mL *In vivo*: 20, 40 mg/kg	Antioxidative Anti-neuroinflammatory	Nrf2-mediated signalling activity
Wang et al. 2017 China	Status epilepticus (SE)	Li-pilocarpine induced SE rat model	5, 10 mg/kg	Enhancing autophagy Anti-apoptotic	Necroptosis proteins (MLKL and RIP-1) and autophagy protein (Beclin-1, LC3BII/LC3BI) expression in the hippocampus
Xue et al. 2008 China	Diabetic encephalopathy (DE)	Streptozotocin-induced DE rat model	5 mg/kg	Anti-apoptotic	Bcl-2 and Bax expression
Xue et al. 2009 China	DE	Streptozotocin-induced DE rat model	0, 1, 5, 10 mg/kg	Antioxidative	lipid peroxide, antioxidant enzymes and NOS activity
Xue et al. 2011 China	–	H_2_O_2_-induced PC12 cells	0.001, 0.01, 0.1, 1 mM	Anti-apoptotic	Bcl-2 and Bax expression, caspase-3 activation, PARP cleavage
Xue et al. 2012a China	–	H_2_O_2_-induced PC12 cells	0.01, 0.05, 0.1, 0.5, 1 mM	Anti-apoptotic	Malondialdehyde, superoxide dismutase, catalase and glutathione peroxidase activity
Xue et al. 2012b China	DE	Streptozotocin-induced DE rat model	1、5、10 mg/kg	Anti-apoptotic	–
Zhu et al. 2018 China	Parkinson’s disease	1-methyl-4-phenyl-1,2,3,6-tetrahydropyridine-induced parkinsonian mice	50mg/kg	Anti-neuroinflammatory; Preserve dopaminergic neurons	Inhibit activation of hippocampal astrocytes and microglia; decrease dopamine and tyrosine hydroxylase levels in the striatum

### Advantages of using AU in treating NDs

First, sources of AU are abundant and inexpensive. In addition, AU is widely distributed in other plants, such as Plantaginaceae, Scrophulariaceae, Oroban-chaceae, Globulariacea, with these plants being widely cultivated in various areas of China (Zeng et al. 2020). Furthermore, cell engineering and callus culture of EUO can be used to extract AU. Second, AU has been shown to possess blood-brain barrier (BBB) permeability. Xue et al. (2015) reported that AU would rapidly distribute in blood-abundant tissues such as the heart, liver, spleen, lungs, kidneys, and brain, with the compound showing a peak concentration 5 min post-dose in the brain, and almost completely disappearing from this organ after 4 h. Its terminal elimination half-life was 1.073 ± 0.241 h, which confirmed its potential for clinical use in the brain, thus warranting further study of its therapeutic efficacy in NDs.

### Research on pharmacological effects of AU in treating NDs

#### Protective effects in nerve cells

Nerve cells include neurons and glial cells, and the loss of neurons is the joint pathological feature of NDs, which may generate to motor, behavioural, and cognitive dysfunctions. Correspondingly, repairing neuronal loss can improve the symptoms of NDs, such as brain trauma, Parkinson’s disease (PD), and epilepsy. Studies suggest that AU protects neurons from damage and increases their numbers by the following mechanisms: first, it inhibits cell hydrogen peroxide (H_2_O_2_)-induced apoptosis (Xue et al. 2011, 2012a). Second, it promotes the differentiation of neural precursor cells (NPCs) into GABAergic neurons (Song et al. 2018b). Third, the survival of neural precursor cells and other specific neuron types such as GABAergic neurons and glutamatergic neurons are enhanced (Song et al. 2018a). Microglia and astrocytes are considered to be important effectors that cause neuroinflammation in the central nervous system (CNS), which activate and release a mass of pro-inflammatory and cytotoxic factors under pathological stimuli, including brain trauma and neurodegenerative diseases, thus aggravating the inflammatory response and causing neuronal dysfunction, in turn triggering or aggravating NDs (Giovannoni and Quintana 2020; Diaz-Castro et al. 2021). Chen et al. (2019) showed that AU significantly reduced Li-pilocarpine-induced activation of hippocampal astrocytes and microglia in epileptic mice. Furthermore, another study manifested that AU treatment reduced 1-methyl-4-phenyl-1,2,3,6-tetrahydropyridine (MPTP)-induced activation of substantia nigra microglia and astrocytes in PD mice, thus confirming the protective effects of AU in the management of NDs (Zhu et al. 2018).

Moreover, synaptic and militated anomalies in the structure and function of neurons are also related to progressive cognitive dysfunction and memory decline, which are the early manifestations of numerous aging-related neurodegenerative diseases (Adalbert and Coleman 2013; Yang et al. 2013; Liu et al. 2019). A study reported that AU induced longer and thicker axons and promoted the regeneration and repair of myelin sheath at 3 weeks in sciatic nerve injury rats (Kim et al. 2014).

#### Protective effects in PD

PD is a neurodegenerative disease, second only to Alzheimer's disease (AD) in incidence, and is characterized by a significant early loss of dopaminergic neurons, primarily in the substantia nigra dense region, as well as other brain regions, such as the locus ceruleus and nucleus basalis of Meynert. Because currently available treatments cannot significantly inhibit the development of this disease, researchers are working to develop effective prevention or treatment (Nakajima and Ohizumi 2019).

A previous study (Zhu et al. 2018) used AU to treat 1-methyl-4-phenyl-1,2,3,6-tetrahydropyridine (MPTP)-induced PD mice and observed behavioural, neurological, and histological changes. Results showed that AU exerted neuroprotective effects and improved the behaviour of the animals. Foremost, MPTP-induced motor deficits and treatment with AU were determined through the transformation of mobility in the pole descent and traction tests. Animals with MPTP-induced motor deficits showed significantly increased descent time and significantly decreased traction scores; however, treatment with AU reversed the aforementioned findings. It was also observed that MPTP significantly reduced the TH-positive neurons in the substantia nigra (SN), and AU significantly reduced the loss of dopaminergic neurons. AU restrained the revitalite of microglia and astrocytes in the SN, thus inhibiting the decrease of tyrosine hydroxylase and dopamine in the striatum of MPTP-induced PD mice.

#### Protective effects in cerebral hemorrhage

In the first few hours after the onset of intracerebral haemorrhage (ICH), severe brain damage is mainly caused by compression and destruction of nearby tissues caused by haematoma formation. Subsequently, inflammation, thrombin activation, and haemolysis caused by primary injury further promote the formation of cerebral edoema, which is associated with poor prognosis and may lead to serious and long-lasting damage (Wang J 2010). Pro-inflammatory mediators released by activated microglia and inflammatory cells which directly induce inflammation or neurotoxicity have also been postulated to contribute to ICH (Ziai 2013). In recent research, scholars from China showed that AU significantly inhibited neurological deficits in rats with cerebral haemorrhage by reducing the expression of interleukin-1β (IL-1β), NF-κB, and tumour necrosis factor alpha (TNF-α) (Liu et al. 2015a, 2015b; Zeng et al. 2020), thus providing a therapeutic role.

#### Protective effects in diabetic encephalopathy

Diabetic encephalopathy (DE) is a diabetic complication characterized by hyperglycaemia, impaired cognitive function, and neurobehavioral deficits. Impaired performance during the Morris water maze and Y-maze tests are characteristic of damaged learning, memory, problem solving, retrieval of learned information, as well as mental and motor speed in rodents with DE. A study (Xue et al. 2012b) showed that AU significantly reduced blood glucose levels and aggrandized body weight, improved depression-like behaviour, and increased neuronal survival in rats with DE. It is speculated that the neuroprotective effects of AU may be achieved by reducing the content of lipid peroxide, regulating the activities of antioxidant enzymes, and decreasing nitric oxide synthase (NOS) activity (Xue et al. 2009). Overall, AU was shown to reverse neuronal apoptosis and eliminate the damage associated with DE (Xue et al. 2008).

#### Protective effects in epilepsy

Epilepsy is one of the most common and disabling chronic neurological disorders, affecting over 70 million people worldwide (Loscher et al. 2020). Epilepsy is characterized by the hyperexcitability of various neuronal circuits that results from the imbalance between glutamate-mediated excitation of voltage-gated cation channels and γ-amino butyric acid (GABA)-mediated inhibition of anion channels, leading to aberrant, sporadic oscillations or fluctuations in neuronal electrical activity (Amtul and Aziz 2017), and may be closely related to deficiencies in processes such as those involving neurotransmitters, ion channels, and cytokines (Solomon et al. 2019). Recently, astrocytes and microglia were found to eliminate most of the extracellular glutamate, which is also related to epilepsy (Wang et al. 2017). A study (Chen et al. 2019) showed that high-dose AU (100 mg/kg) reduced the intensity of seizures, lengthened the incubation period of seizures, reduced the mortality of mice, inhibited the activation and hypertrophy of astrocytes and microglia in the CA1 and CA3 regions of the hippocampus of mice with Li-pilocarpine induced epilepsy, increased the content of GABA in the hippocampus of these animals, and decreased their glutamate levels. In addition, another study (Wang et al. 2017) showed that neuronal apoptosis increased significantly in the hippocampus of epileptic rats and hippocampal neuronal cell death was a significant reason that was postulated to promote the occurrence and development of epilepsy. Furthermore, pre-treatment with AU was found to relieve the damage in Li-pilocarpine-induced status epileptic animals by inducing autophagy and inhibiting programmed cell necrosis, reducing the number of apoptotic neurons, and increasing the number of surviving neurons. This effect was hypothesized to have been achieved by autophagy induction and necroptosis inhibition (Wang et al. 2017).

#### Protective effects in anxiety and depression

The World Health Organization (WHO) reported that there were more than 322 million people with depression worldwide in 2017, and this number has been increasing. Therefore, effective and low toxicity drugs are urgently needed to manage this condition. A previous study in rodents (Chu et al. 2020) indicated that AU possesses anxiolytic effects, as was demonstrated by mice showing a higher percentage of time spent in open arms and open arm entries during an elevated plus maze test and more time spent in the light area of the apparatus used in the light/dark box tests. In addition, AU has antidepressant activity, as revealed by reduced immobile time of rodents in the forced swimming and tail suspension tests. To date, the pathophysiological mechanism of depression is understood to include a variety of hypotheses, with the monoamine hypothesis in the classic theory being the most widely recognized. The aforementioned study evaluated AU-treatment related changes by quantifying the concentration of dopamine, noradrenaline, serotonin, glutamate, and GABA in the brains of mice by high-performance liquid chromatography with fluorescence detection, with the authors deducing that there were conspicuous decreases in the levels of glutamate and noradrenaline, increased levels of serotonin and GABA, but no changes in the concentration of dopamine were observed.

#### Protective effects in traumatic brain injury

An increasing number of traumatic brain injuries (TBIs) occur with the development of society, and consist of protopathic and secondary brain injuries, with oxidative stress (OS) and inflammation being thought as vital pathobiological symbols of secondary brain damage. A study (Wang et al. 2020) explored the potential function of AU in TBI and showed that the compound significantly attenuated brain edoema and histological damage, improved learning and memory functions, and promoted neuronal survival in a mouse model of TBI. Overall, the consequences of the study suggested that the neuroprotective effect of AU in TBI may be attributed to inhibiting OS and inflammatory responses.

### Molecular mechanisms of AU in treating NDs

#### Antioxidative effects

The brain is the main target of excessive oxidative damage due to its high content of polyunsaturated fatty acids (Cobley et al. 2018). A previous study verified the antioxidant effect of AU *in vivo* and *in vitro* and found that AU increased NOS production or inhibited generation of reactive oxygen species (ROS), decreased MDA and NO content, and increased SOD, CAT, GSH, GSH-Px, HMOX1/2, and catalase levels (Xue et al. 2009; Li et al. 2020); however, the antioxidant effect was not observed in normal PC12 cells (Xue et al. 2012a). Furthermore, the potential molecular mechanisms of these salutary effects are involved in the regulation of ROS or NOS by triggering Nrf2-ARE (Wang et al. 2020), nNOS/NO (Yang et al. 2018), Nrf2/HO-1 (Shen et al. 2019) and β3-adrenoceptor/AC/cAMP signalling pathways (Wu et al. 2018), as well as decreasing thioredoxin interaction protein (TXNIP) levels (Duan et al. 2019). AU enhances the translocation of Nrf2 into the nucleus, an effect that is dependent on AMPK activation. Suppression of AMPK levels may inhibit Nrf2 activation, which was identified following the elimination of suppressive effects of AU by the HO-1 inhibitor ZnPP and AMPK inhibitor pre-treatment (Qiu et al. 2018). In addition, AU can decrease the activation of MAPK pathways (Li et al. 2021).

#### Anti-neuroinflammatory effects

Neuroinflammation is a complicated process orchestrated by a diverse population of glial cells in the CNS and peripheral immune cells. It is a pivotal participant in various neurological disorders (Yang and Zhou 2019), and increases the production and aggregation of Aβ, phosphocreatine Tau protein, destroys the BBB, and accelerates the degeneration and death of neurons (Varatharaj and Galea 2017). Studies have shown that AU reduces the levels of TH, Iba-1 (microglia marker), and GFAP (astrocyte marker) (Zhu et al. 2018), and was reported to significantly inhibit pro-inflammatory cytokines (HMGB1, RAGE, TLR4, MyD88, and NF-κB p65, TNF-α, IL-1β, IL-6) (Shen et al. 2019; Wang et al. 2020; Zhang et al. 2020; Li et al. 2021), the mechanisms involved in inactivating the NLRP3/ASC/caspase-1 inflammasome (Duan et al. 2019), the HMGB1/TLR-4/NF-κB signalling pathway (Zhang et al. 2020), as well as activating the Keap1/Nrf2 (Gao et al. 2021) or AMPK/Nrf2 signalling pathways (Qiu et al. 2018).

#### Enhancing autophagy in the nervous system

Autophagy commonly accelerates the clearance of denatured proteins to prevent further development of disease in the early stages of NDs. As the disease progresses, protein accumulation increases, triggering the continuous activation of autophagy and inducing cell death. Moreover, impaired function of lysosomes by abnormal proteins in NDs, such as mutant huntingtin protein (mHTT) and tau, inhibits the elimination of abnormal proteins, eventually causing nerve cell death and hindering the function of the nervous system (Button et al. 2015). Studies have found that AU upregulates the expressions of Beclin-1, LC3B, and p-AMPK by activating AMPK signalling to enhance autophagy; these effects were partially reversed when AMPK was knocked down (Wang et al. 2017; Yue et al. 2021).

#### Anti-apoptotic effects

Apoptosis is a typical form of programmed cell death and a precise process regulated by genes, which is considered to be the final common pathway of cell death in tumours, epilepsy, ischaemia (local and global), trauma, and neurodegenerative diseases. A series of cysteine proteases called caspases (Savitz and Rosenbaum 1998; Shalini et al. 2015), activated cysteine proteases, are responsible for degrading important proteins in the cells, thereby causing cell apoptosis (D'Arcy 2019). Moreover, the proteins involved in the apoptosis pathway mainly include caspases, adaptor proteins, Bcl-2, and the IAP protein family. An existing study showed that AU remarkably inhibited the overexpression of Bax and necroptosis proteins (MLKL and RIP1), as well as the cleavage of caspase-3, PARP, and caspase-9, but enhanced Bcl-2 expression (Xue et al. 2008; Wang et al. 2017; 2019).

## Conclusions

**Figure 2.**
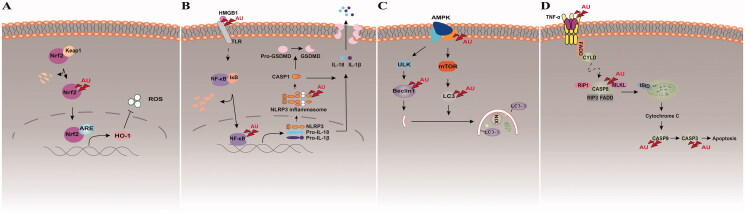
Therapeutic targets of AU in treating NDs. Studies have shown that AU can treat NDs through its anti-oxidation (A) and anti-neuro-inflammation (B) properties, as well as inducing autophagy (C) and anti-apoptosis (D). The red triangle points represent the targets of AU in NDs discovered in existing studies.

AU exerts a protective effect in NDs by enhancing autophagy or attenuating injury induced by OS, apoptosis, inflammation, and neurotransmitter disorders. Existing studies have explored the effects of AU in treating PD, AD, ICH, DE, epilepsy, anxiety, and depression, as well as TBI, with animals and cells being the subject/models used for the investigations. The pharmacological mechanisms involved in repairing neuronal loss include increasing GABA in the synapse, promoting differentiation of neural precursor cells into GABAergic neurons, promoting antioxidant and anti-neuroinflammatory effects, as well enhancing autophagy and anti-apoptotic actions. The therapeutic mechanisms and targets of AU in treating NDs are demonstrated in Figure 2. This review summarizes studies that highlighted the role of AU in protecting against NDs and its postulated mechanisms, thus underscoring it as a promising resource for use in future clinical applications.

However, there are some limitations or shortcomings to the use of the reviewed molecule: A growing body of evidence suggests that AU possesses neuroprotective effects; nonetheless, the therapeutic targets have only been elucidated *in vivo* in animals or cells but not in humans. In addition, the therapeutic effects of AU in other NDs remain to be explored, and a systematic approach mechanism to the application of the compound should be established.

## Data Availability

Data sharing is not applicable to this article as no new data were created or analysed in this study.
